# Diffuse B Cell Non-Hodgkin's Lymphoma Presenting Atypically as Periprosthetic Joint Infection in a Total Hip Replacement

**DOI:** 10.1155/2017/7195016

**Published:** 2017-05-24

**Authors:** Aysha Rajeev, Angela Ralte, Nameer Choudhry, Faizan Jabbar, Paul Banaszkiewicz

**Affiliations:** ^1^Department of Trauma and Orthopaedics, Queen Elizabeth Hospital, Gateshead Health NHS Foundation Trust, Sheriff Hill, Gateshead NE9 6SX, UK; ^2^Department of Pathology, Queen Elizabeth Hospital, Gateshead Health NHS Foundation Trust, Sheriff Hill, Gateshead NE9 6SX, UK

## Abstract

The occurrence of extranodal primary B cell non-Hodgkin's lymphoma is rare. Total hip replacement is one of the most common orthopaedic procedures performed. There has been an increased incidence of primary lymphomas involving periprosthetic sites. Chronic inflammation due to metal debris arising from the prosthetic implants has been evidenced as one of the causes for the development of soft tissue lymphomas albeit rarely. We describe a case report of a 77-year-old patient who had underwent a cemented total hip replacement in the past who further developed large B cell primary non-Hodgkin's lymphoma. She presented initially with signs and symptoms highly suggestive of underlying periprosthetic infection. The radiological imaging was also indicative of periprosthetic infection. The diagnosis was eventually confirmed after an open biopsy. This case underlines the importance of considering and including soft tissue malignancy in the differential diagnosis of suspected chronic periprosthetic infection.

## 1. Introduction

Primary non-Hodgkin's lymphoma (NHL) is a rare malignancy of the musculoskeletal system and is known to affect up to 5–25% of patients [1, 2]. Within the joint it can either affect the synovium [3] or directly involve the intra-articular tissue [1]. The patients often present with joint pain if the bone is affected primarily [4]. It is important to appreciate that lymphomas can involve extra-articular tissue and potentially mimic infection [5]. It has also been shown that patients with inflammatory arthritis have a higher incidence of hematopoietic cancers especially after joint replacement surgery [6, 7]. Diffuse B cell NHL usually affects patients above 60 years of age and is known to be a very aggressive tumour [8].

We describe a unique and rare case report of a patient who presented with signs and symptoms suggestive of periprosthetic joint infection after total hip replacement, which finally concluded to have been diffuse B cell non-Hodgkin's lymphoma.

## 2. Case Presentation

A 77-year-old woman presented to our Accident and Emergency department with a one-year history of a painful right hip replacement. She complained of difficulty in weight bearing and a gradually diminishing walking distance such that in the four weeks prior to admission she was essentially housebound. Furthermore she reported her hip pain had progressed to an extent that it was present at rest and on occasion woke her up from sleep at night.

She had been reviewed six months previously by her local GP who had started her on high dose oral steroids for a provisional diagnosis of polymyalgia rheumatic under the guidance of the rheumatologist. There was no history of trauma.

Her past medical history included osteoarthritis, iron deficiency anemia, and a right cemented Exeter total hip replacement performed three years previously.

On examination she appeared generally unwell and frail. She was apyrexial with no evidence of any localized features of erythema, swelling, redness, or skin changes around the affected hip. There was severe tenderness on palpation around the greater trochanter. Her active range of motion of the right hip was limited to 20° flexion with all other movements being severely restricted. Passive range of motion was also limited.

**Figure 1:**
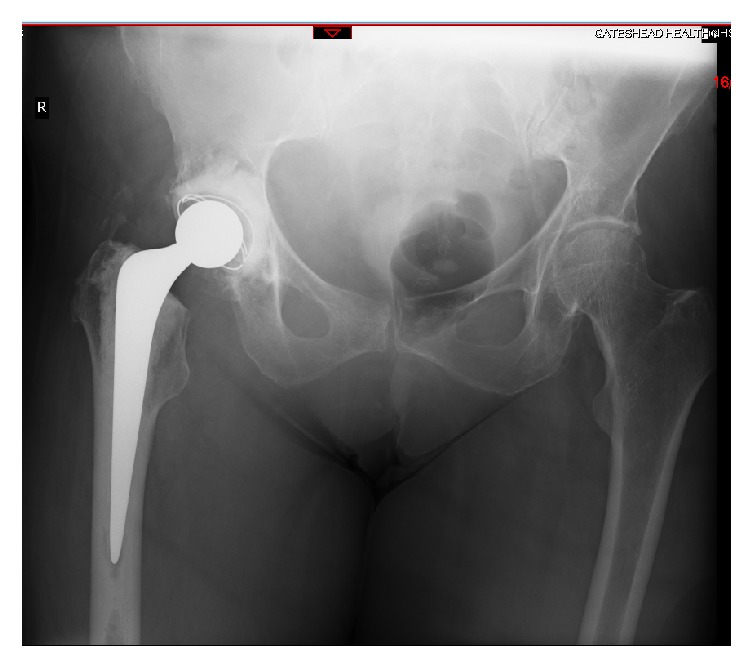
Plain X-ray of the right hip with normal soft tissues and satisfactory position of prosthesis.

**Figure 2:**
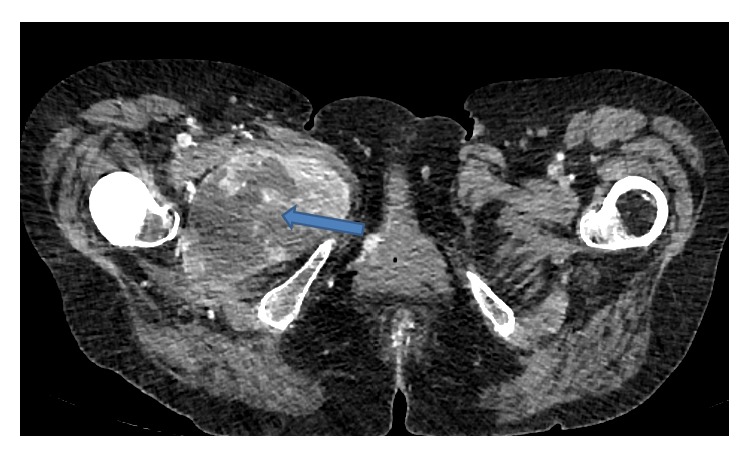
CT scan of the pelvis showing a cystic lesion in close proximity to the femoral stem suggesting an inflammatory pathology.

**Figure 3:**
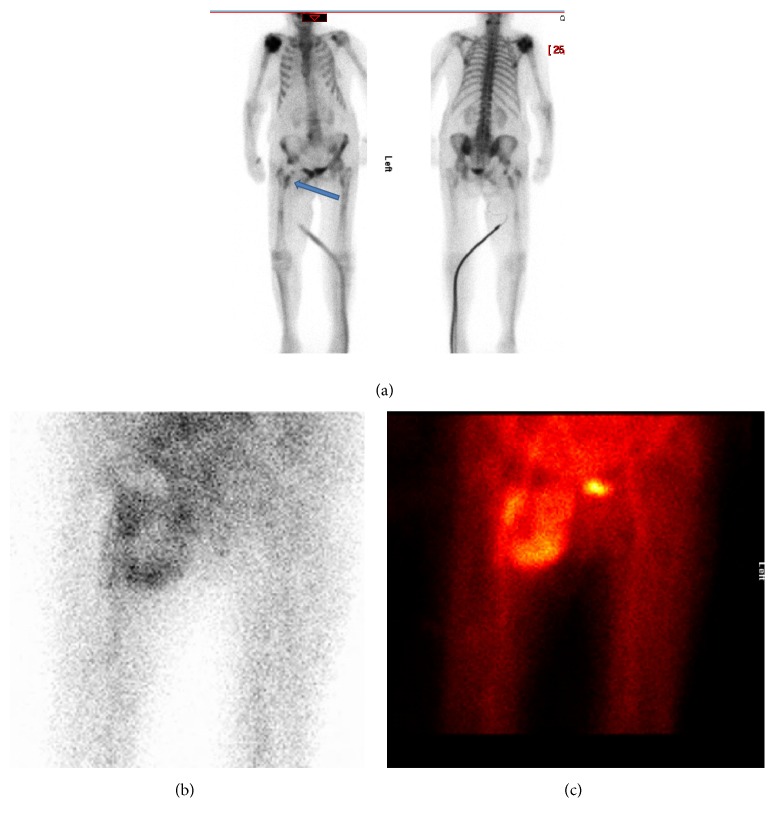
(a) Te^99^ bone scan showing increased uptake suggestive of infection. (b and c) The hypervascular abnormality on the early images would be in keeping with a soft tissue abscess with a necrotic centre, possibly related to infection of the right hip prosthesis.

**Figure 4:**
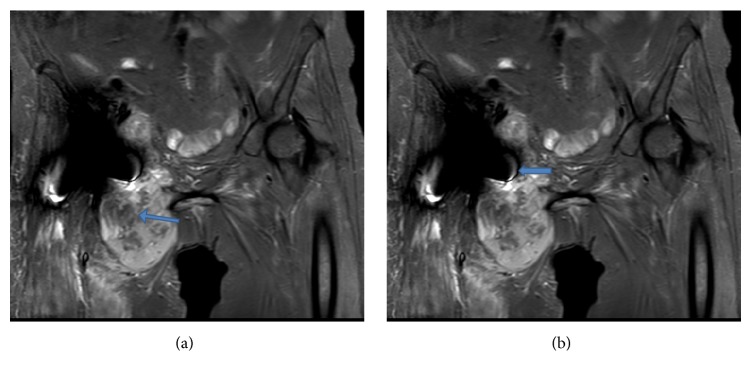
(a) T2 weighted images showing a lesion with central liquefaction necrosis. (b) T2 weighted images showing the close proximity of the lesion to the medial side of the prosthesis with effusion into the joint.

The blood results showed a haemoglobin of 86 with an elevated WCC 32.5, CRP 246, and ESR 100. Plain X-rays of the hip were normal (Figure 1) and further cross-sectional CT imaging demonstrated a cystic mass closely related to the hip prosthesis suggesting an inflammatory pathology (Figure 2). The bone scan using Te^99^ and SPECT (Single Photon Emission Computed Tomography) showed a large an area of intense hypervascularity at the medial aspect of the right proximal femur (Figures 3(a), 3(b), and 3(c)). The MRI scan revealed huge lesion with solid components and areas of liquefaction necrosis involving the periarticular hip region (Figures 4(a) and 4(b)). In view of the fact that the patient has got clinical signs and symptoms of sepsis and the imaging investigations pointing towards and infective pathology a provisional diagnosis of periprosthetic hip infection was made.

**Figure 5:**
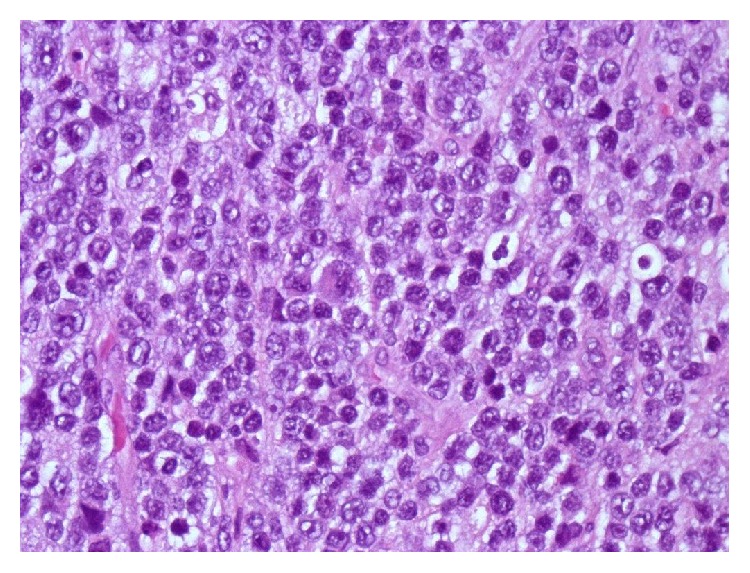
Medium sized atypical lymphoid cells with large vesicular nuclei and prominent nucleoli, some showing a hint of plasmacytoid differentiation.

**Figure 6:**
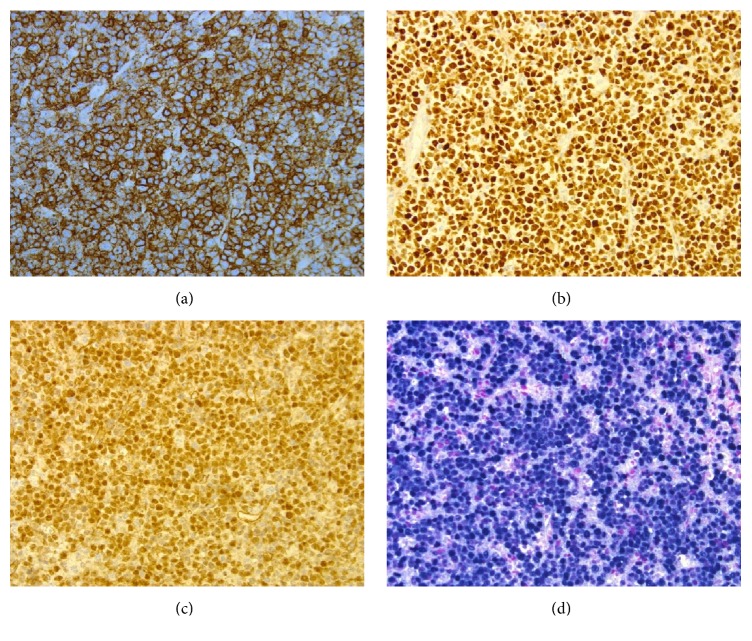
Immunostains showing positivity with CD138 (a), MUM 1 (b), PAX 5 (c), and EBV ISH (d) confirming diffuse large B cell lymphoma.

A hip aspiration was performed in theatre under image intensifier guidance with strict aseptic conditions. The aspirate revealed slightly turbid fluid. The following results including Gram stain, culture, and sensitivity were negative. Postaspiration intravenous antimicrobials were commenced. However the patient continued to remain unwell despite antimicrobial therapy with a progressively increasing WBC and CRP count. The decision was therefore taken to explore and perform open biopsy of the pelvic collection and soft tissue mass. At surgical exploration, a 4 cm × 6 cm cavity was identified. Deep tissues were noted to be oedematous, necrotic, and inflamed, but macroscopically they were not typical findings in keeping with an abscess cavity. Specimens were sent for histology and microbiology. All direct culture and sensitivity specimens from the hip biopsy were negative. Histopathology of the specimen demonstrated sheets of atypical lymphoid cells with a hint of plasmacytoid differentiation (Figure 5). Immunostains showed the neoplastic cells to express CD138, MUM 1, PAX5, and EBV ISH (Figures 6(a), 6(b), 6(c), and 6(d)). They demonstrated patchy weak expression of CD56 and CD30. They were negative for CD20, CD79, CD19, cyclin D1, ALK, HHV8, CD2, CD3, CD4, CD5, CD8, CD33, CD43, EMA, Cytokeratin, and S-100. In situ hybridization and immunostaining for immunoglobulin heavy and light chains appeared polytypic and was difficult to interpret. Therefore a PCR based analysis of B cell clonality was performed which showed clonal IG Kappa gene rearrangements consistent with clonal B cell population. In the context of a metallic hip replacement, the features were those of diffuse large B cell lymphoma with chronic inflammation.

Due to clinical condition of the patient and poor prognosis, in liaison with the haematological team who advised that the patient was in the terminal phase of her illness, appropriate management would be palliation. The patient was transferred to a hospice for continuing care and support and the patient passed way after only two weeks of admission to hospice.

## 3. Discussion

Non-Hodgkin's lymphoma (NHL) is a malignant proliferation of either B or T cell lymphocytes in a disorderly fashion. Diffuse large B cell lymphoma is the most common of all lymphomas accounting for about 35% [15]. The distribution of these tumours is predominantly nodal, but up to one-third of all NHL can be extranodal [16].

Any part of the musculoskeletal system can be affected in lymphomas and can present as a soft tissue mass resembling either infection or soft tissue tumour [5, 17]. The soft tissue involvement is more common in NHL rather than Hodgkin's lymphoma [18]. There are several theories put forward suggesting the cause of primary soft tissue lymphomas. One such hypothesis suggests that chronic inflammation involving the soft tissue such as inflammatory connective tissue disorders, viral infections, and local debris from prosthetic joint replacement implants can damage the cell membrane of macrophages. This in turn leads to the release of proinflammatory mediators which stimulate the immune system responses [19].

There are several studies which looked at increased incidence of lymphoma after total hip replacement. Gillespie et al. concluded that in the first decade following total hip replacement the incidence of tumours of the lymphatic and haemopoietic systems was significantly greater and this may be as the resultant effect of the prosthetic implants [6]. Another study by Visuri and Koskenvuo concluded that patients with a total hip replacement have an increased risk of lymphoma and leukaemia and also suggested the role of chrome-cobalt-molybdenum alloy in carcinogenesis [20]. The association of primary NHL and total hip replacement has been described in various case reports in the literature (Table 1).

Usually diffuse B cell NHL affects patients above the age of 60 years and predominantly occurs in males with a ratio of 1.5 : 1 [8, 21]. The systemic symptoms such as weight loss and pyrexia are usually absent [17]. The case described in this report also did not show any systemic symptoms. The incidence of involvement of the proximal femur (27–34%) and the pelvis (15–17%) has been reported [22, 23]. The imaging investigations including CT and MRI scan can describe the morphology of the lesion, but a definitive diagnosis of B cell NHL is only possible by an open biopsy [24]. Interestingly in our patient the signs, symptoms, and imaging studies performed were all highly suggestive of a periprosthetic infection, but open biopsy results concluded otherwise.

One of the differential diagnoses of mass lesions after total hip replacement is a metal-on-metal pseudotumour, also known as aseptic lymphocyte-dominant vasculitis-associated lesion (ALVAL), especially around a metal-on-metal hip or knee replacement [25]. They are large focal solid or semiliquid masses around the hip prostheses. The pseudotumours mimic features of neoplasia or infection. The principal symptom is pain and there may be restricted range of movements of the hip joint. The incidence of symptomatic pseudotumours following metal-on-metal hip arthroplasty is in the region of 5% and females are commonly affected [26, 27]. Metal-on-metal pseudotumours are sterile inflammatory lesions. Excessive wear is considered the initiating process, leading to the release of nanometer sized particles. These are cytotoxic to macrophages once phagocytised and may lead to necrosis within the lesions. They are usually associated with high serum and joint fluid cobalt and chromium ion levels [28].

The other cause of psuedotumours after THR is avulsion of abductor tendons associated with fluid collection. MRI scans are diagnostic with pure fluid signals and the anatomical location of the pseudotumour [29].

The treatment of diffuse B cell NHL is combined chemotherapy and radiotherapy with good outcomes [30]. The five-year survival rate with combined modality therapy is 88% [22].

## 4. Conclusion

Primary diffuse large B cell NHL is a rare musculoskeletal malignancy. It can present atypically as a periprosthetic joint infection; therefore clinicians should have a high index of clinical suspicion to consider and include tumour pathology in the differential diagnosis.

In cases of suspicion of alternate pathology one should consider an open biopsy for a timely diagnosis and treatment.

## Figures and Tables

**Table 1:** Case studies with diffuse B cell NHL after total hip replacement.

Case study	Age/gender	Duration of symptoms	Presenting symptoms	Treatment
Syed et al. [9], 2002	75/F	7 years	Thigh and groin pain with inability to weight bear	Radiotherapy
Radhi et al. [10], 1998	64/F	4 years	Pain and swelling in the thigh	Chemotherapy and radiotherapy
Ito and Shimizu [11], 1999	80/F	8 years	Painful hip	Radiotherapy
Ganapathi et al. [12], 2001	85/M	14 months	Chronic discharging sinus	Died before treatment
O'Shea et al. [13], 2006	75/F	13 years	Chronic discharging sinus, pain, and swelling of thigh	Chemotherapy, radiotherapy, and excision arthroplasty
Hsieh et al. [14], 2007	30/F	4 years	Painful hip	Chemotherapy
Rajeev et al., 2016 (current study)	72/F	3 years	Painful hip	Died
